# Integrated Mapping of Establishment Risk for Emerging Vector-Borne Infections: A Case Study of Canine Leishmaniasis in Southwest France

**DOI:** 10.1371/journal.pone.0020817

**Published:** 2011-08-09

**Authors:** Nienke Hartemink, Sophie O. Vanwambeke, Hans Heesterbeek, David Rogers, David Morley, Bernard Pesson, Clive Davies, Shazia Mahamdallie, Paul Ready

**Affiliations:** 1 Department of Farm Animal Health, Theoretical Epidemiology, Faculty of Veterinary Medicine, Utrecht University, Utrecht, The Netherlands; 2 Georges Lemaître Centre for Earth and Climate Research, Earth & Life Institute, Université Catholique de Louvain, Louvain-la-Neuve, Belgium; 3 Spatial Ecology and Epidemiology Research Group, Department of Zoology, Oxford, United Kingdom; 4 Department of Parasitology, Université de Strasbourg, Strasbourg, France; 5 London School of Hygiene and Tropical Medicine, London, United Kingdom; 6 Department of Entomology, Natural History Museum, London, United Kingdom; Institute for Animal Health, United Kingdom

## Abstract

**Background:**

Zoonotic visceral leishmaniasis is endemic in the Mediterranean Basin, where the dog is the main reservoir host. The disease's causative agent, *Leishmania infantum*, is transmitted by blood-feeding female sandflies. This paper reports an integrative study of canine leishmaniasis in a region of France spanning the southwest Massif Central and the northeast Pyrenees, where the vectors are the sandflies *Phlebotomus ariasi* and *P. perniciosus*.

**Methods:**

Sandflies were sampled in 2005 using sticky traps placed uniformly over an area of approximately 100 by 150 km. High- and low-resolution satellite data for the area were combined to construct a model of the sandfly data, which was then used to predict sandfly abundance throughout the area on a pixel by pixel basis (resolution of c. 1 km). Using literature- and expert-derived estimates of other variables and parameters, a spatially explicit *R*_0_ map for leishmaniasis was constructed within a Geographical Information System. *R*_0_ is a measure of the risk of establishment of a disease in an area, and it also correlates with the amount of control needed to stop transmission.

**Conclusions:**

To our knowledge, this is the first analysis that combines a vector abundance prediction model, based on remotely-sensed variables measured at different levels of spatial resolution, with a fully mechanistic process-based temperature-dependent *R*_0_ model. The resulting maps should be considered as proofs-of-principle rather than as ready-to-use risk maps, since validation is currently not possible. The described approach, based on integrating several modeling methods, provides a useful new set of tools for the study of the risk of outbreaks of vector-borne diseases.

## Introduction

Zoonotic visceral leishmaniasis (ZVL) is a parasitic disease of humans, caused by the protozoan *Leishmania infantum*. In Southern Europe, this parasite is transmitted by female sandflies (Diptera: Phlebotominae) of the subgenus *Larroussius* [1]–[5] and primarily causes disease in dogs and other canids. Due to the high prevalence in domestic dogs in many areas in the Mediterranean region (prevalences up to 34% have been reported in Spain and up to 20% in France [6]) and the high case fatality ratio ([7]), canine leishmaniasis constitutes a considerable veterinary problem [6]. Infected dogs also act as a reservoir host for the human disease; sandflies infected by dogs may, during a later blood meal, infect humans. Healthy humans are dead-end hosts for ZVL and do not usually develop any symptoms, but children and immuno-suppressed people may develop serious symptoms when infected, especially if malnourished [3] or co-infected with HIV [8]. Changes in climate and other environmental factors, such as land use, could lead to further expansion of the areas where canine leishmaniasis can be sustained, by increasing the range or seasonal abundance of the sandfly vectors, or by influencing other aspects of the transmission cycle [5]. Presence of the vector is not the only factor determining whether or not a pathogen can establish. Even if the vector is abundant, the values of other factors may result in a situation where introduction of the pathogen does not lead to a large outbreak. Such factors are often environmentally determined, and include the replication rate of the pathogen, the vector biting rate, host availability, or the infectious life span of either vectors or hosts. We therefore need a tool to predict whether or not canine leishmaniasis can establish after introduction in a certain area and under certain climatic and environmental conditions. Such a tool is available in the form of the basic reproduction number (*R*_0_) of the disease, defined as the expected average number of secondary cases caused by one infectious individual placed in a naïve population. The value of *R*_0_ is a measure of the likely success of invasion into a population. If it is higher than 1, an outbreak of the disease is possible; if it is smaller than 1, the disease will die out [9], [10]. *R*_0_ can be seen as a quantity integrating, in a properly weighted way, all factors determining whether or not a pathogen can establish in a given area. Previous studies have presented approximations of *R*_0_ for canine leishmaniasis for endemic situations; the approximations are based on epidemic data such as prevalence [11] and age at which dogs acquire the disease [12]. However, such approximations can only be used in endemic areas, and are therefore not suitable for studying whether or not the disease can establish in a new area. Also, such approximations do not allow for scenario studies, since the influence that climatic or land use factors may have on essential aspects of the transmission process (vector and host populations, transmission parameters and the temperature-dependency of vector activity and survival) are not taken into account.

In this paper, we develop a method to predict the risk of establishment of canine leishmaniasis in new geographic locations and present a map that depicts the spatially varying values of *R*_0_ over part of southwest France. Extensive field work was carried out in this area on the two regional sandfly vectors of canine leishmaniasis: *Phlebotomus (Larroussius) ariasi* Tonnoir, 1921 and *P. (L.) perniciosus* Newstead, 1911. The area shows variation in the prevalence of canine leishmaniasis, the eastern part having more cases than the western part [13].

In order to construct such an *R*_0_ map, we need to derive an expression for *R*_0_ and parameterize this expression for each pixel in the map. Sandfly densities have to be estimated for all pixels, based on results from intensive sampling in the target area. To do this, we used a statistical model based on a combination of high- and low-resolution remotely sensed data. The high resolution imagery provided important details of land-scape features (such as land use and habitat fragmentation) that were not detected by the low resolution imagery which recorded habitat seasonality and climate. Previously, low-resolution analysis had been combined with process-based models in a study of bluetongue virus in the Netherlands [14] but, to our knowledge, the combination of high- and low-resolution satellite data is novel. The resulting vector abundance predictions were, together with a dog density map and several literature-based parameter estimates, used as inputs for a process-based temperature-dependent *R*_0_ model. This model is used to create an *R*_0_ map for canine leishmaniasis by using a geographic information system (GIS) to combine all the information.

## Materials and Methods

### Terminology

The terms “sandfly” and “sandflies” will be used for statements that apply to both of the regional vectors, *P. perniciosus* and *P. ariasi*. Species names will be mentioned only if a statement refers specifically to them.

### Expression for R*_0_*

First, we derived an expression for *R*_0_ for canine leishmaniasis, using the next-generation matrix (NGM) [10] approach. The NGM method provides a framework for derivation of *R*_0_ for disease systems that involve more than one type of infected individual (referred to as type-at-infection [15]). For vector-borne diseases, there are at least two types-at-infection, the vector and the host, but the principle can also be applied to more complex disease systems [16]. The elements of this matrix, k_ij_, represent the expected number of cases of type-at-infection *i* caused by one individual of type-at-infection *j*. For canine leishmaniasis, we define two types-at-infection - the sandfly (type 1) and the dog (type 2) - resulting in the following NGM:
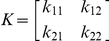
where

k_11_ = mean number of sandflies infected by one infected sandfly
k_12_ = mean number of sandflies infected by one infected dog
k_21_ = mean number of dogs infected by one infected sandfly
k_22_ = mean number of dogs infected by one infected dog

**Figure 1:**
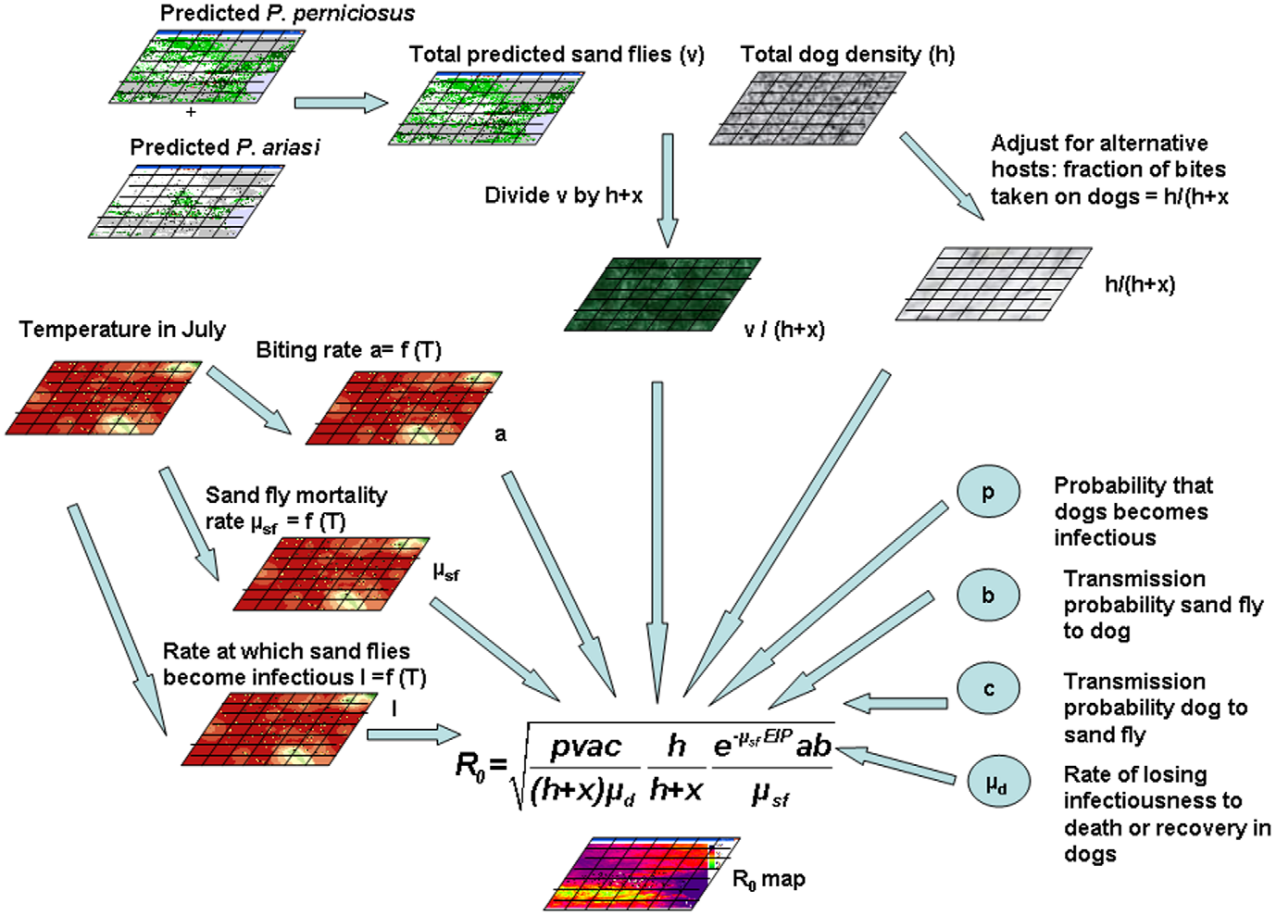
Schematic overview of the approach.

**Figure 2:**
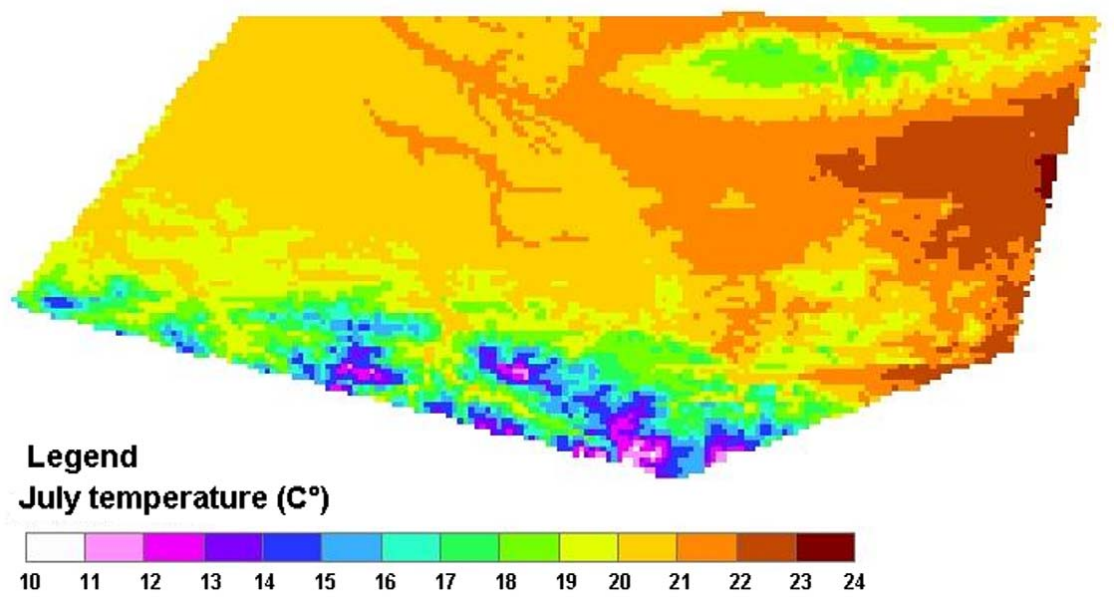
Temperature map of the study region: average temperature in July.

**Figure 3:**
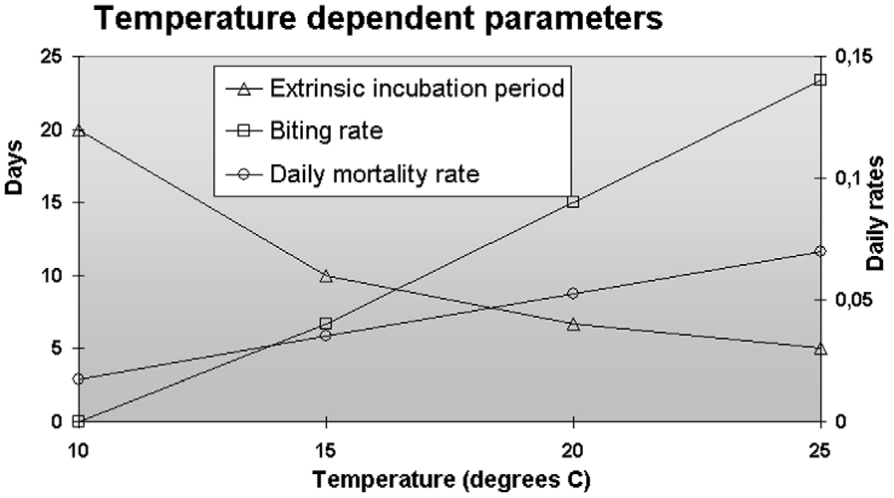
Relationship between temperature and known temperature-dependent parameters.

The dominant eigenvalue of the NGM has been shown to equal *R*_0_ [10], [17]. Assuming no direct transmission between dogs or between sandflies (i.e., *k_11_* and *k_22_* are zero) we only have to derive expressions for element *k_12_* (the mean number of sandflies infected by one infected dog) and element *k_21_* (the mean number of dogs infected by one infected sandfly). First, we will derive an expression for element *k_12_*, the mean number of sandflies infected by one infected dog. A newly infected dog will develop infection (become infectious) with probability *p*, and it will infect sandflies with a probability *c* per bite for as long as it stays infectious. The duration of the infectious period, which might end either due to recovery (spontaneous or as a result of treatment) or due to the death of the dog, is denoted as 1/*μ_d_*, the reciprocal of a combined ‘death or recovery’ rate. The biting rate is denoted by *a* (the reciprocal of the length of the gonotrophic cycle, as sandflies normally take one blood meal per oviposition cycle [18]). The regional vectors are opportunistic feeders, with host choice being related to the availability of individual host species [19], [20], rather than to their specific attractiveness. Sandflies are known to feed on humans, canids, equines, bovids (cattle/sheep) and birds [19]–[22], many of which are dead-end hosts for leishmaniasis. The sandfly density is denoted by *v*, the dog density by *h* and the alternative host density by *x*. Assuming that the ‘burden’ of biting sandflies (*av*) is spread evenly over the entire population of hosts, the number of bites per night for a dog will be *av*/(*h*+*x*). The number of sandflies infected by one newly infected dog will then equal:
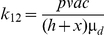
The average number of dogs infected by one infected sandfly, *k_21_*, is derived as follows. In order to become infective, each newly infected sandfly has to survive the extrinsic incubation period (or EIP, the interval between the acquisition of an infectious agent by a vector and the vector's ability to transmit the agent to other susceptible vertebrate hosts), the proportion doing so being exp^(−μsf EIP)^. Each infected sandfly bites at rate *a*, and transmits the infection with probability *b* per bite for the rest of its lifespan (determined by the sandfly mortality *μ_sf_*). We assume that a fraction *h/(h+x)* of the blood meals is taken from dogs. The average number of dogs infected by one infected sandfly will then equal:
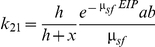
The NGM will therefore equal:
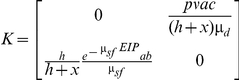
The dominant eigenvalue of this NGM is the expression for R_0_:
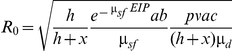
The next step in constructing the R_0_ map is to parameterize this expression. A schematic overview of the approach is given in Figure 1. For four parameters (transmission efficiencies, *b* and *c*, the probability of a dog becoming infectious, *p*, and the rate of losing infectiousness, *μ_d_*), the values were assumed to be constant over space and we used point values and ranges obtained from the literature (see Table 1). For parameters known to vary with temperature, such as the biting rate *a*, sandfly mortality *μ_sf_* and duration of the EIP, we used simple, linear models to describe them (see Table 1 and Supporting Information S1). Using 1 km resolution average July daytime temperatures (for the period 2000–2005) from the WorldClim website (www.worldclim.org, assessed April 2009), the value of each of the temperature-dependent parameters was calculated for each pixel (Figure 2 and 3). In the absence of information indicating otherwise, all vector-related parameter estimates were taken to be identical for the two vector species.

**Table 1:** Parameters: point estimates and ranges.

	Description	Point estimate	Ranges used for sensitivity analysis	Sources
c	Transmission efficiency from dog to sandfly	0.7	0.5–0.9	[37]–[42]
b	Transmission efficiency from sandfly to dog	0.01	0.005–0.05	None
p	Probability of a dog becoming infectious	0.5	0.2–0.8	[7], [12], [39], [43]–[45]
μ_d_	Rate of a dog losing infectiousness, either because of treatment, self-cure or death	0.02	0.01–0.04	Average lifespan of infected dogs is two years [7], [41], but only part of that period overlaps the sandfly season
x	Alternative host (not dog) density	5	1–10	x is estimated so that h/(h+x) varies between ½ [19] in low dog density areas and almost 1 in high dog density areas (settlements), where most bites are on dogs
a	Sandfly biting rate	(T-14)/100+0.03	0.03–0.16	[34], [46] *
EIP	Extrinsic incubation period	100/(T-5)	3–20	[42], [47], [48] *
μ_sf_	Sandfly mortality	0.0035 (T-5)	0.017–0.07	[39], [49] *

Estimates are based on experiments/observations on *P. ariasi* [34], [39], [49], *P. perniciosus* [37], [38], [40], [42], or both [46].

*For information on the derivation of these values see the Supporting Information S1.

Dog density and the sandfly density both vary over space in a complex manner, because they depend on many different factors. In the following sections, we explain in some detail how we estimated the values for each of these parameters for each pixel.

### Predicting dog density

The dog density map, with values of *h* for each pixel, was constructed by the data management team of the EDEN project. The basic principle underpinning the approach is that known dog population data obtained from the Facco website (http://www.facco.fr, asssessed in 2003) are related to 1 km resolution human population densities obtained from the SEDAC website (http://beta.sedac.ciesin.columbia.edu/gpw/, assessed in 2003) that have been summarised at a (sub-) national level, using information on dog-ownership in different categories of agglomerations. This relationship is then used to predict dog density at 1 km resolution using a high spatial resolution grid of human population density. The estimated dog density ranges from 0 to 778 dogs per km^2^ in the study area, with a mean density of 7.6 dogs per km^2^.

### Predicting sandfly abundance

Sandfly abundance predictions were based on surveys of the study area carried out by the joint NHM and Strasbourg team in July 2005. July was chosen because it was found to be the summer month when both vectors were abundant. The study area, situated between latitude 42°20′N and 43°40′N and between longitude 0°E and 3°E, encompassed the forested southwest foothills of the Massif Central (the Montagne Noire), the forested northeast foothills of the eastern Pyrenees mountains and the intervening lower ground that has a land cover containing more settlements and arable crops [23]. The altitude of collection sites ranged from 96.8 to 811 metres above sea level as measured by GPS. Sandflies were sampled at 169 sites, at each of which a single sticky trap (an A4 paper soaked in castor oil) was placed in each of 5–20 drainage holes of a roadside retaining wall. Sampling was synchronised, with papers put out over 4 days (10–13^th^ July) and collected in the same order 4 days later (14–17^th^ July). There were no extreme weather events in this period. The mean numbers of males of each vector species per paper (the sample unit) were the model input data. Some trap sites recorded zero catches, and these were used in the models to represent sandfly absence. Sites were excluded from the analysis if less than 5 traps were recovered.

The statistical model developed to predict sandfly abundances used two sorts of satellite data; high resolution (30 m pixels) LANDSAT TM data and low resolution (1 km pixels) Terra MODIS and other data. The LANDSAT data were those previously described and used by Martínez *et al.* [23] and the MODIS data and their processing are described by Scharlemann *et al.* [24]. The high resolution imagery, which had previously been classified into land-cover classes (see Table 1 in Martinez *et al.* [23]), was used to provide several predictor variables reflecting land-cover and composition that were thought to be important for sandflies. These derived variables included shape (as documented by the Shape Index in FRAGSTATS [25]) and size of forest patches and crop patches (aggregated crop classes), the distance between forest patches (‘proximity’), the proportion of the surrounding area occupied by urban areas, several crops, pasture, coniferous, deciduous and sclerophylous forests and other types of vegetation, and the Shannon's diversity index as defined by Forman [26]. Spatial variables (i.e. those referring to a spatial rather than point measure of landscape features) were calculated within a 1000 m buffer zone centred on each sandfly sampling site. Hence, such spatial variables, that essentially recorded sub-1 km pixel resolution details of land-cover, were finally consolidated into 1 km resolution pixels that coincided in size with those of the coarser MODIS sensor pixels. MODIS data, through temporal Fourier processing, [27], [28] capture elements of habitat seasonality (not available from the LANDSAT imagery) that are also felt to be important in sandfly seasonal dynamics. The MODIS imagery included ‘middle infra-red’ (MIR), daytime Land Surface Temperature (LST), night-time LST, Normalised Difference Vegetation Index (NDVI) and Enhanced Vegetation Index (EVI). Additional low resolution data used in the models were CMORPH data from the CPC website (http://www.cpc.noaa.gov/products/janowiak/cmorph_description.html, assessed May 2009) and WORLDCLIM rainfall data from the WorldClim website (http://www.worldclim.org, assessed May 2009), also temporal Fourier processed, and the digital elevation layer (DEM) distributed with MODIS v5 data. Thus the combination of high- and low- resolution imagery provided a wide range of both spatial and seasonal details of habitat characteristics, for use in the statistical models describing the spatial variation in sandfly abundance across the study area. In total, the models had available to them 27 variables derived from LANDSAT and 71 MODIS and other low-resolution image variables (ten Fourier variables for each of the seven sensor or other multi-temporal data, and one for the DEM).

The suite of predictor variables was used in a non-linear discriminant analysis (NLDA) framework where the predicted variable was the mean recorded sandfly abundance at each site [27], [28]. NLDA works on categorical rather than continuous variables, so the full range of sandfly abundance for each species was divided up into three abundance classes (boundaries determined to give approximately equal numbers of observations in each category); for each species there were also two absence classes (determined by clustering some of the environmental data for the absence sites, using the k-means clustering algorithm). There was therefore a total of five classes to be described and distinguished by each model.

In the NLDA modeling, predictor variables were selected in a step-wise inclusive manner to minimize Akaike's Information Criterion corrected for the number of variables used in the model (AICc) [29]. No more than ten predictors were selected, and all were used in the models for each species since the AICc values were lowest with ten variables (indicating that more variables could have been included to improve the models further). Stepwise inclusive methods tend to select variables in sequence that are un-correlated with the variables already in the analysis, because variables that are highly correlated with those already in the predictor variable set are less likely to improve model fit than those that are not. The end result is a set of predictor variables that tends to be the least inter-correlated. We emphasize that this methods selects a set of variables that together predict the data best, not a set of ten best predicting individual parameters. The variables selected for each species are listed in Table 2.

**Table 2:** Predicting variables that performed best in the final model.

	Rank	1/AICc	AICc	Description of variable
***P. ariasi***	1	0.006	179.5	Mean shape index of Crops
	2	0.006	166.28	DEM
	3	0.006	153.9	Nighttime LST phase 1
	4	0.007	144.86	Nighttime LST amplitude 3
	5	0.008	126.6	NDVI phase 2
	6	0.008	117.75	Proportion of crops: sparsely vegetated
	7	0.010	104.96	EVI phase 1
	8	0.010	96.92	Daytime LST amplitude 3
	9	0.012	83.01	WORLDCLIM precipitation phase 3
	10	0.015	68.01	Proportion of pasture
***P. perniciosus***	1	0.005	192.42	Proportion of crops: vineyards
	2	0.006	180.57	Daytime LST phase 2
	3	0.006	174.13	EVI amplitude 1
	4	0.006	166.72	NDVI amplitude 2
	5	0.006	159.45	Proportion of complex cultivation pattern
	6	0.007	143.15	NDVI amplitude 3
	7	0.008	129.46	EVI phase 3
	8	0.009	109.16	EVI variance
	9	0.010	98.42	WORLDCLIM precipitation minimum
	10	0.012	83.76	CMORPH precipitation phase 1

The resulting models were then applied throughout the study area to make predictions of the sandfly abundance category for each pixel, dependent upon values of the key predictor variables for that pixel; the result is therefore a ‘risk map’ for the abundance of sand-flies. Some pixels had conditions more extreme than any in the data used to create the models in the first place (the ‘training set’); such pixels were classified as ‘no prediction possible’ in the output imagery and could not therefore be used further. For all the remaining pixels the predictions were used to estimate the mean total abundance of sandflies per km^2^. This was done by multiplying the predicted mean abundance (the average of the upper and lower limit of each abundance category) by a factor f, where f was given values of 500, 1000 and 5000 in different output R_0_ maps, to acknowledge the great uncertainty of this factor and to quantify its influence. More details of how these values have been estimated can be found in the Supporting Information S2.

### Construction of R*_0_* maps

The actual *R*_0_ map was constructed by combining all the vector abundance prediction maps, the dog density map and all the relevant parameters in a Geographic Information System. The dog density map, the predicted vector abundance maps and the temperature maps were overlaid in ArcGIS 9.3, and the values at set 1 km pixel intervals were retrieved by using an Intersect Point Tool (part of Hawth's Analysis Tools, downloaded from http://www.spatialecology.com/htools/isect.php on 28 May 2009). The intersect point tool was used because the raster pixels did not always exactly coincide in the different sorts of imagery used, hence precluding the use of more direct, raster-based calculations. The extracted data were processed using the R_0_ expression derived above and the parameter estimates given in Table 1. Using a feature-to-raster conversion, the values of *R*_0_ were then converted to a raster layer. Obviously, the *R*_0_ value could be calculated only for pixels for which a prediction for the sandfly species had been made. Pixels with ‘no prediction’ for either sandfly species obtained a ‘no prediction’ value in the *R*_0_ maps.

### Sensitivity analysis

Unavoidably, there is a large amount of uncertainty in most parameter estimates. The effect of this uncertainty on the outcome was assessed, using Latin Hypercube sampling. Instead of using point estimates, we sampled from a prescribed range of possible values for each parameter. For the parameters in Table 1, we used the ranges noted in the table, whereas for the sandfly abundance prediction map, the value of each pixel was allowed to be one category higher or lower than predicted. As stated above, the multiplication factor f was allowed to vary between 500 and 5000. The dog density (h) was allowed to vary from two times lower up to two times higher than the original prediction. For each parameter, 1000 values were sampled, using Latin Hypercube sampling. The value of *R*_0_ was calculated for each of the 1000 sets of parameter values, yielding 1000 values of *R*_0_ for each pixel. The analysis was performed using the statistical package R with an additional lhs package available on the R website (www.cran.r-project.org, assessed in April 2009). For each pixel, the mean and the 5% and 95% percentiles of the 1000 values were calculated and exported to ArcGIS.

## Results

### Sandfly abundance prediction maps

**Figure 4:**
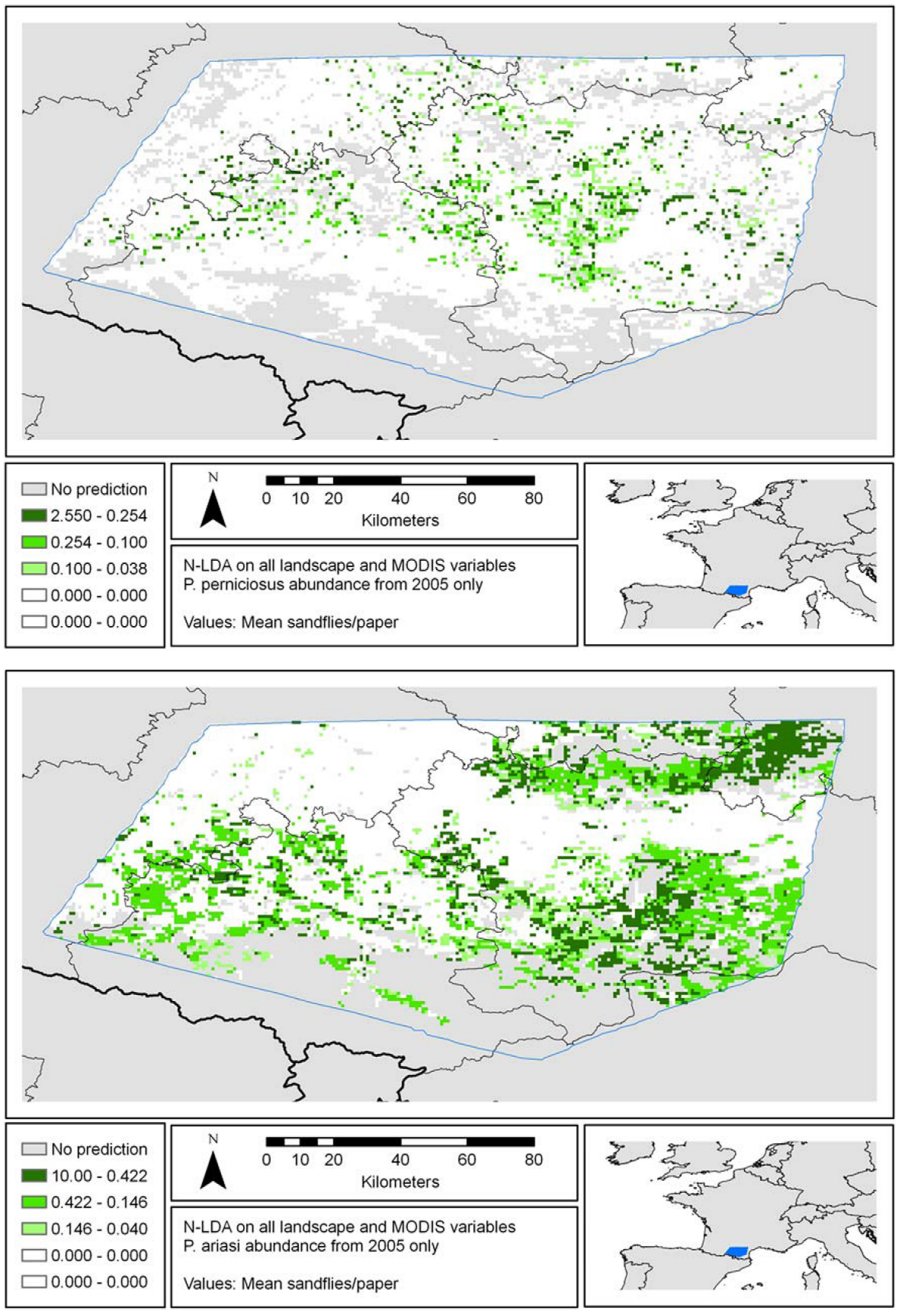
Predicted.abundance of *P. perniciosus* (upper panel) and of *P. ariasi* (lower panel), based on the integrated model.

The final NLDA model was used to predict the abundance of each species for each pixel (Figure 4). We also compared the performance of this model with other NLDA models produced using only the LANDSAT-derived variables or only the low-resolution MODIS and other variables. The sensitivity and specificity of the final model were slightly better than the model based only on low resolution variables for *P. ariasi*, the predominant species. Both the final and low resolution variable models outperformed the high resolution variable model for each species according to three measures (Cohen's Kappa, sensitivity and specificity), except for the specificity of the *P. perniciosus* model (see Supporting Information S3 for more details).

For *P. ariasi*, the final model selected three landscape (high resolution) variables and seven low resolution variables (see Table 2). The predicted abundance categories were 0.04–0.146, 0.146–0.422 and 0.422–10.0. The accuracy, as judged by Cohen's Kappa (0.8110±0.0732) was ‘excellent’ according to Congalton's classification of kappa values (κ<0.4, poor; 0.4<κ<0.75, good; and κ>0.75, excellent). Final model sensitivity and specificity both exceeded 0.90.

For *P. perniciosus* the final model selected two landscape variables (proportion of vineyards and of complex cultivation pattern) and eight low resolution variables (including daytime temperature, vegetation indexes and precipitation, see also Table 2). The predicted abundance categories for this species were 0.038–0.1, 0.1–0.254 and 0.254–2.55. The accuracy was again excellent (Cohen's kappa of 0.7749±0.0790), and, once again, model sensitivity and specificity both exceeded 0.9 (better for sensitivity but worse for specificity compared with the *P. ariasi* model).

### R*_0_* maps

**Figure 5:**
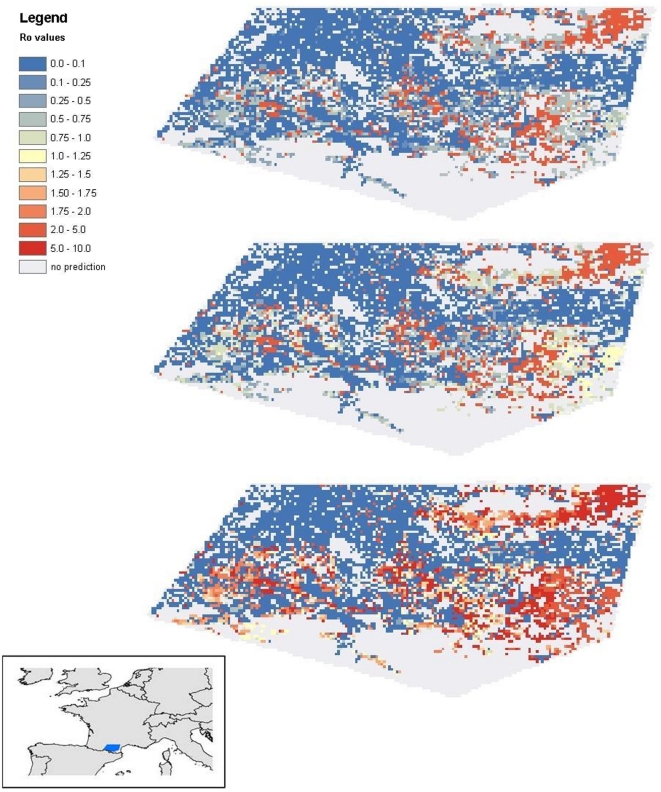
*R*_0_ maps based on the predicted vector abundance maps, the dog density map, and the parameter point estimates in  Table 1 . *R*_0_ maps are depicted for different values of the multiplication factor: f = 500 (a), f = 1000 (b), and f = 5000 (c). Resolution is 1 km^2^.

*R*_0_ maps were constructed based on the parameter estimates in Table 1, the temperature map and the predicted vector abundance maps and dog density maps. The *R*_0_ value was calculated for different values of the multiplication factor used to translate predicted numbers of sandflies per paper into vector densities per km^2^ (Figure 5). The overall pattern in the *R*_0_ maps shows comparatively high values in the northeast and the southeast of the study area, lower values in between and very low values (often zero) in the northwestern part, and this is clearly dictated by the pattern of the sandfly abundance predictions, especially that of the *P. ariasi* distribution, which was captured in higher numbers than *P. perniciosus*, and thus is most important in determining the variation in the value of *v.* Different values for the multiplication factor change the values for *R*_0_ but not the pattern.

### Sensitivity analysis

**Figure 6:**
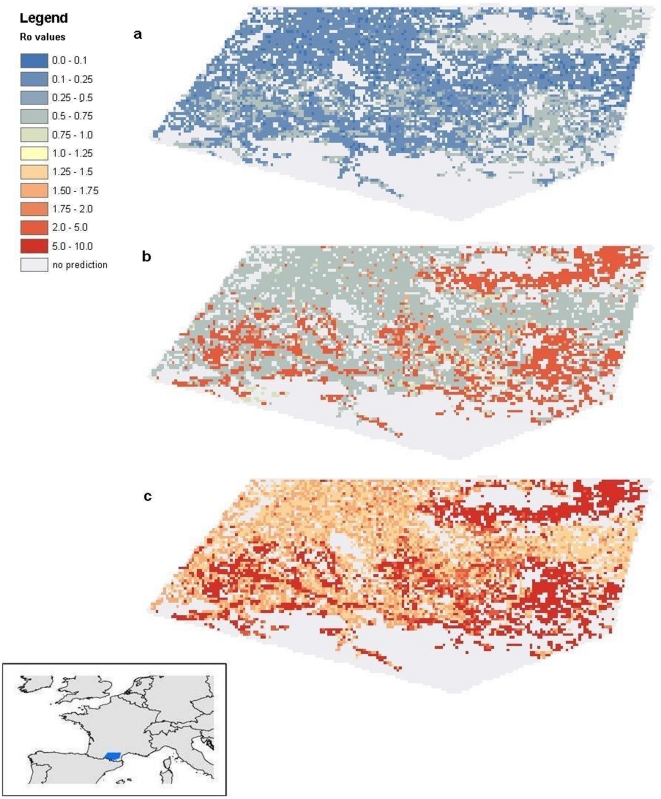
*R*_0_ maps resulting from sampling from parameter ranges with the Latin Hypercube sampling method. Uniform sampling from the ranges in Table 1 yielded 1000 different sets of parameter values and hence 1000 values of *R*_0_ per pixel. The 5% percentile of the 1000 values is depicted in a map (a), as well as the mean values (b) and the 95% percentile, (c). Resolution is 1 km^2^.

The sensitivity analysis yielded a range of 1000 *R*_0_ values for each pixel. One map was constructed based on the mean values (Figure 6b), and two other maps reflect the 5 percentile (Figure 6a) or the 95 percentile (Figure 6c) predicted limits of *R*_0_. Given the range of parameter values therefore, the value of *R*_0_ is likely to be higher than the value in Figure 6a and lower than the value in Figure 6c.

## Discussion

This paper presents a detailed description of the construction of an R_0_ map for canine leishmaniasis. Its novelty lies in the combination of a variety of low and high resolution satellite data to reflect different aspects of the natural environment of sandflies in the study area. Land cover type and details of landscape features, such as the size and shape of different vegetation patches are best captured by LANDSAT imagery. Seasonality can only be captured by multi-temporal data of the sort represented by MODIS and the other seasonal data used. A single algorithm then selected the appropriate combinations of low- and high-resolution data to make predictions of the abundance classes of the two sandfly species present in the study area. These predictions then fed into a biological model predicting R_0_ values throughout the area. Other recent studies have used similar approaches to study the risk of malaria re-emergence in Lower Saxony in Germany [30] and the risk of bluetongue outbreaks in Switzerland [31] and the Netherlands [14]. However, these studies were not based on variables measured at different spatial scales.

Concerning the results of the integrated prediction model, several conclusions can be drawn from this study. *P. ariasi* appears to predominate in the northeast and southeast of the study area in the forested foothills, whereas *P. perniciosus* is predicted to be more abundant in the intervening areas at lower altitudes (Figure 4). This is consistent with previous observations [32]. It seems that *P. ariasi* can survive in colder habitats than *P. perniciosus* [33] (temperature is closely linked to altitude).

The field data were collected using a standardized sampling method in a short period of time encompassing no extreme weather events. The number of sandflies collected and the fraction of females depend heavily on local circumstances, including the weather, and sandfly trapping results are known to show considerable variation seasonally and between years [18], [22], [32], [34]. Additional data from the same location for a number of years would help quantify such variability, but the larger the area the greater will be the environmental variation and the more difficult it will be to synchronise catches throughout it. Models of the sort used here can allow for greater environmental variability (captured by the satellite imagery) but not yet for the seasonal variation of catches at any one site. The fact that the field work was performed in one month only, also implicates that the risk map applies primarily to the situation in July. Since sandflies are not known to fly for long distances, it is unlikely that the distribution in July is very different from distributions in less favorable months, a notion supported by the fact that the two vectors had such distinct altitudinal patterns of abundance. It is however quite likely that the abundance is higher than in other months. This would mean that the presented maps may tend to overestimate the risk, but given the long infectious period in the dog, one favorable period in the year could be sufficient to let a transmission cycle persist. Methods to more accurately estimate persistence would need input for vector-abundance throughout the year.

In the *R*_0_ model, a number of simplifying assumptions had to be made. Firstly, direct transmission between dogs, that might happen occasionally [5], was not considered in the model. We can assess the effect of this simplification by including a hypothetical dog-to-dog reproduction number of 0.1 in element k_22_ (i.e. assuming a ten percent probability that a dog infects another dog, which is much higher than expected in the field), revealing that this has little effect on R_0_. We can then consider two extreme cases: areas with no sandflies, and areas with high sandfly abundance. In the first case, the system is reduced to a one-host system and the R_0_ value is 0.1, hence well below unity. In areas of high sandfly abundances, R_0_ is high and the relative increase of R_0_ due to dog-to-dog transmission is small. Only where R_0_ is just below unity, does dog-to-dog transmission make any real difference to disease persistence.

Another simplification arose from the fact that the number of alternative sources of blood meals for the sandflies was unknown. Hence, we had to assume a fixed density of alternative hosts throughout the area. In reality, the abundance of alternative hosts will depend on the feeding habits of the sandfly species concerned, and its habitat characteristics. Even though foxes, badgers and cats have been suggested to play a role in the transmission cycle in France, Portugal and Spain [5], [32], [35], there is insufficient information on their role in the spread of leishmaniasis to include them in the present model.

The biting rate (a) is assumed to equal the reciprocal of the gonotrophic cycle, but infected sandflies may actually bite more frequently, because a *Leishmania* parasite-induced ‘plug’ in their midgut [36] may prevent them from feeding fully.

More generally, the parameter estimates are best guesses, especially for the temperature-dependency relationships and for the transmission efficiency from sandfly to dog; more field and laboratory research is therefore needed in these areas. Also, the method of translating (predicted) numbers of male sandflies sampled on a sticky paper into real biting densities for female sandflies needs to be validated and improved.

The R_0_ maps presented here should be considered as proofs-of-principle rather than as ready-to-use risk maps. The patterns of the *R*_0_ values in the final model are largely determined by the vector abundance predictions, because many parameters do not vary too much over space. We would expect very low *R*_0_ values in the colder regions to the northeast and south of our study area, because the higher sandfly survival at low temperatures will be counteracted by the lower biting rate and the longer EIP. However, all these higher altitude regions (>800 m a.s.l.) had a “no prediction” result, because there were no high altitude sites in the training set data used to create the models.

Validation of the maps presented here is currently not possible, due to the lack of dog prevalence data at the resolution of the study area. An attempt was made to extend the model to a much larger area of France (where canine leishmaniasis prevalence data are available, but only at département level), but was unsuccessful because the environmental variation in the training set was limited compared with that of the rest of France.

The biological *R*_0_ risk mapping approach has numerous advantages over more statistically-based risk mapping approaches. First, both the quantity of *R*_0_ and the parameters involved have clear biological and epidemiological interpretations. Second, the processes involved are modelled mechanistically, which allows us to gauge the effect of changing values of various biological determinants on the risk of an epidemic following introduction of the pathogen. By using temperature dependent parameters, the outcome of the oft-opposing effects of temperature on transmission parameters can be studied. This method, at least theoretically, is capable of identifying areas where the vector can persist but the pathogen cannot establish. A drawback of the method is the fact that it requires estimates for many different parameters. When these parameters cannot be estimated, only in endemic situations the *R*_0_ approximations based on epidemiological data [11], [12] can be a useful alternative, but the latter do not have the advantages of our approach. To our knowledge, this is the first analysis that combines a vector abundance prediction model, based on remotely-sensed variables measured at different levels of spatial resolution, with a fully mechanistic process-based temperature-dependent *R*_0_ model. We therefore suggest that our approach provides a useful new set of tools for the study of the risk of outbreaks of vector-borne diseases.

## Supporting Information

Supporting Information S1 **Derivation of the temperature-dependent parameters.**
(DOC)

Supporting Information S2 **Translating the predicted number per trap into vector density.**
(DOC)

Supporting Information S3 **Predicting the sandfly density.**
(DOC)
