# Supplementary material for: Integrated Mapping of Establishment Risk for Emerging Vector-Borne Infections: A Case Study of Canine Leishmaniasis in Southwest France
Source: PLoS One. 2011 Aug 9;6(8):e20817. doi: 10.1371/journal.pone.0020817 (PMC3153454; doi:10.1371/journal.pone.0020817)
Supplement: Supporting Information S1 — Derivation of the temperature-dependent parameters. (DOC) [file pone.0020817.s001.doc]

**Supporting Information S1**

**Derivation of the temperature-dependent parameters**

Biting rate *a*

Sandflies are assumed to be active at temperatures between 14 and 28 C° [1, 2]. The biting rate (i.e. the reciprocal of the interval between the blood meals) is thought to be no higher then 0.16 (corresponding to a six-day interval between blood meals) [3]. For this study, we used the algorithm *a* = (T-14)/100+0.03 for all pixels with a temperature of 14 C° or higher; pixels with a lower temperature have a value of zero for the biting rate. The biting rate in the non-zero pixels ranges from 0.03 to 0.12, corresponding to intervals between blood meals of 8 to 33 days.

Duration of the EIP

The development of the parasites is faster at higher temperatures, hence the duration of the EIP is shorter. The EIP of *L. infantum* has been reported to last from a few (3-7) days [4] up to 20 days [5]. We chose the algorithm EIP = 100/(T-5), so that the duration of the EIP ranges from 5.5 days (at 23.1 C°) via 10 days (at 15 C°) to 20 days (at 10 C°) in the study area.

Mortality rate (µ_sf_)

The survival of *Phlebotomus* species is restricted to a temperature range of 5-30 C° [2, 6]. The life span of female sandflies is approximately 2 weeks to 2 months, and it is known to be longer at lower temperatures and higher humidity [6, 7]. Based on this notion, we approximated the relationship between the daily mortality of adult sandflies and temperature by µ_sf_ = 0.0035(T-5).
