# Supplementary material for: Integrated Mapping of Establishment Risk for Emerging Vector-Borne Infections: A Case Study of Canine Leishmaniasis in Southwest France
Source: PLoS One. 2011 Aug 9;6(8):e20817. doi: 10.1371/journal.pone.0020817 (PMC3153454; doi:10.1371/journal.pone.0020817)
Supplement: Supporting Information S2 — Translating the predicted number per trap into vector density. (DOC) [file pone.0020817.s002.doc]

**Supporting Information S2**

**Translating the predicted number per trap into vector density**

This is done using literature information on the number of sandflies biting a dog per night, the biting rate, *a,* and the local dog density. Based on experiments with dog-baited traps [1, 2], we know that in endemic areas the number of bites per dog could be of the order of tens or even hundreds per night. This number theoretically equals the biting rate (*a*) times the sandfly density (*v*) divided by the dog density. We deduced that in a situation where a dog is bitten one hundred times per night, in an area with an average dog density of 8/km^2^ and biting rate of 0.15 per day, *v* would have to be around 5000, meaning that the obtained predictions should be multiplied by a factor of 500 up to 5000. We used several values for this multiplication factor (referred to as “f”) in the analysis, to acknowledge this uncertainty and to quantify its influence. These calculations make no assumptions about the abundance of alternative hosts. The more of these there are, and the more they are used by sand-flies, the higher should be the multiplication factor relating sandfly catches to sandfly abundance per square kilometer.

References

1.     Rioux JA, Golvan YJ. (1969) Épidémiologie des leishmanioses dans le sud de la france. Paris: Institut National de la Santé et de la Recherche Médicale. 223 p.

2.     Killick-Kendrick R, Rioux JA. (2002) Mark-release-recapture of sand flies fed on leishmanial dogs: The natural life-cycle of *Leishmania infantum* in *Phlebotomus ariasi*. Parassitologia 44 (1-2): 67-71.
