# Supplementary material for: Integrated Mapping of Establishment Risk for Emerging Vector-Borne Infections: A Case Study of Canine Leishmaniasis in Southwest France
Source: PLoS One. 2011 Aug 9;6(8):e20817. doi: 10.1371/journal.pone.0020817 (PMC3153454; doi:10.1371/journal.pone.0020817)
Supplement: Supporting Information S3 — Predicting the sandfly density. (DOC) [file pone.0020817.s003.doc]

**Supporting Information S3**

**Predicting the sandfly density**

The final NLDA model was compared to NDLA models using only high resolution or only low resolution variables. It turned out that sensitivity and specificity of the final model were slightly better than the NLDA model based only on low resolution variables for *P. ariasi* and this was the predominant species. Both the integrated and low resolution only models outperformed the model based on high resolution variables for each species according to three measures (Cohen’s Kappa, sensitivity and specificity), except for the specificity of predicting the abundance of *P. perniciosus*.

| *P. ariasi* | High resolution variables | Low resolution variables | Both |
| --- | --- | --- | --- |
| Cohen’s Kappa | 0.6044 + 0.0979 | 0.841 + 0.083 | 0.8110 + 0.0732 |
| Sensitivity | 0.7222 | 0.897 | 0.9155 |
| Specificity | 0.9310 | 0.942 | 0.9882 |
| Corrected Akaike Information Criterion (AICc) | 240.1 | 86.1 | 68.0 |

| *P. perniciosus* | High resolution variables | Low resolution variables | Both |
| --- | --- | --- | --- |
| Cohen’s Kappa | 0.6791 + 0.0953 | 0.863 + 0.082 | 0.7749 + 0.0790 |
| Sensitivity | 0.5745 | 1.000 | 0.9574 |
| Specificity | 0.9537 | 0.914 | 0.9182 |
| Corrected Akaike Information Criterion (AICc) | 145.1 | 79.2 | 83.8 |
